# Supplementary material for: Triploidy in zebrafish larvae: Effects on gene expression, cell size and cell number, growth, development and swimming performance
Source: PLoS One. 2020 Mar 2;15(3):e0229468. doi: 10.1371/journal.pone.0229468 (PMC7051096; doi:10.1371/journal.pone.0229468)
Supplement: S1 Table — (DOCX) [file pone.0229468.s001.docx]

**Supporting information**

| **Date** | **Mode of fertilization** | **Egg number** | **Shock temperature**  **(°C)** | **Shock duration (min)** | **Time post fertilization***  **(min)** | **Survival 24h**  **(%)** | **Number tested** | **Triploidy efficiency**  **(%)** |
| --- | --- | --- | --- | --- | --- | --- | --- | --- |
| 2017/12/08 | natural | 57 | 41 | 2 | 2.5 | 57.9 | 15 | 6.7 |
| 2017/12/08 | natural | 39 | 41 | 3 | 2.5 | 87.2 | 15 | 13.3 |
| 2017/12/08 | natural | 13 | 41 | 4 | 2.5 | 38.5 | 3 | 0 |
| 2018/01/11 | natural | 87 | 41 | 2 | 2.5 | 51.7 | 36 | 80.6 |
| 2018/01/11 | natural | 121 | 41 | 2.5 | 2.5 | 74.4 | 20 | 55.0 |
| 2018/03/09 | ivf | 157 | 0.5 | 10 | 2.5 | 11.5 | 1 | 0 |
| 2018/03/16 | ivf | 105 | 4 | 10 | 2.5 | 53.3 | 12 | 0 |
| 2018/03/16 | ivf | 100 | 4 | 20 | 2.5 | 37.0 | 12 | 58.3 |
| 2018/03/30 | ivf | 333 | 4 | 20 | 3 | 77.2 | 12 | 100 |
| 2018/08/27 | ivf | 366 | 4 | 20 | 3 | 70.5 | 12 | 100 |
| 2018/12/06 | ivf | 110 | 4 | 20 | 3 | 69.1 | 33 | 93.9 |
| 2019/01/24 | ivf | 198 | 4 | 20 | 3 | 63.1 | 36 | 100 |
| 2019/02/07 | ivf | 136 | 4 | 20 | 3 | 74.3 | 39 | 97.4 |
| 2018/03/30 | ivf | 330 | - | - | - | 76.7 | - | - |
| 2018/08/27 | ivf | 275 | - | - | - | 84.7 | - | - |
| 2018/12/06 | ivf | 87 | - | - | - | 77.0 | - | - |
| 2019/02/07 | ivf | 112 | - | - | - | 70.5 | - | - |

**S1 Table: Triploidy induction efficiency**
