# Supplementary material for: Triploidy in zebrafish larvae: Effects on gene expression, cell size and cell number, growth, development and swimming performance
Source: PLoS One. 2020 Mar 2;15(3):e0229468. doi: 10.1371/journal.pone.0229468 (PMC7051096; doi:10.1371/journal.pone.0229468)
Supplement: S1 Appendix — (DOCX) [file pone.0229468.s002.docx]

**S1 Appendix. Analysis of responders per startle.**

**Results Pearson’s χ^2^ tests.**

**Startle 1:**

| **Ploidy level \ Response type** | **Responder** | **Non-responder** | **Total** |
| --- | --- | --- | --- |
| **# 2n larvae** | 69 | 2 | 71 |
| **# 3n larvae** | 41 | 12 | 53 |
| **Total** | 110 | 14 |  |

*χ^2^* = 10.01, *df* = 1, *p* = 0.0016

**Startle 2:**

| **Ploidy level \ Response type** | **Responder** | **Non-responder** | **Total** |
| --- | --- | --- | --- |
| **# 2n larvae** | 57 | 14 | 71 |
| **# 3n larvae** | 22 | 31 | 53 |
| **Total** | 79 | 45 |  |

*χ^2^* = 18.09, *df* = 1, *p* < 0.001

**Startle 3-10:**

| **Ploidy level \ Response type** | **Responder** | **Non-responder** | **Total** |
| --- | --- | --- | --- |
| **# 2n larvae** | 332 | 236 | 568 |
| **# 3n larvae** | 84 | 340 | 424 |
| **Total** | 416 | 576 |  |

*χ^2^* = 147.27, *df* = 1, *p* < 0.001
