# Supplementary material for: Triploidy in zebrafish larvae: Effects on gene expression, cell size and cell number, growth, development and swimming performance
Source: PLoS One. 2020 Mar 2;15(3):e0229468. doi: 10.1371/journal.pone.0229468 (PMC7051096; doi:10.1371/journal.pone.0229468)
Supplement: Attachment — Submitted filename: Response to reviewers_20200205.docx [file pone.0229468.s004.docx]

ACADEMIC EDITOR COMMENTS TO THE AUTHOR

Academic Editor

Comments to the author:

The reviewer requested that the caveat attached to Table S1 should really appear in the main text somewhere. I think it would be fine to just move this text into the relevant part of the Discussion. This is a very minor point, but as some readers may never actually look a the supplemental table I agree its probably a good idea.

Reply: We thank you for this suggestion. We have now included this information in the Discussion section of the manuscript (line 518-523).

REVIEWER’S COMMENTS TO THE AUTHOR

Reviewer 1

Comments to the author:

All comments have been addressed.

Reviewer 2

Comments to the author:

The authors have satisfyingly responded to my specific comments, except for

one which is listed below and can be addressed through clarification in the text.

Table S1 – In response to a previous comment (comment #3) the authors added a disclaimer at the end of Table S1 that the timing of fertilization was determined by visually assessing egg release of females during natural fertilization and that this may result in timing irregularities . However, in the body of the text the authors suggest only that in “these studies slightly different conditions were applied”. Considering the authors compare the efficiency of their method to others for heat shock, it should be clearly mentioned in the text that natural fertilization was used for the heat shock studies and that this may result in some timing irregularities, which as they mention strongly affects the success of triploidy induction.

Reply: Indeed, we agree we should mention this caveat in the main text. Therefore, we included this information to the Discussion section of the manuscript (line 518-523).
